# Brief Metacognitive Therapy for Emotional Distress in Adult Cancer Survivors

**DOI:** 10.3389/fpsyg.2019.00162

**Published:** 2019-01-31

**Authors:** Peter L. Fisher, Angela Byrne, Louise Fairburn, Helen Ullmer, Gareth Abbey, Peter Salmon

**Affiliations:** ^1^Department of Psychological Sciences, University of Liverpool, Liverpool, United Kingdom; ^2^Liverpool Clinical Health, Royal Liverpool and Broadgreen University Hospital NHS Trust, Liverpool, United Kingdom; ^3^Psychology Service, Royal Liverpool and Broadgreen University Hospital NHS Trust, Liverpool, United Kingdom

**Keywords:** cancer, survivors, emotional distress, metacognitive therapy, open trial

## Abstract

**Background:** Adult cancer survivors often experience substantial psychological morbidity following the completion of acute cancer treatment. Unfortunately, current psychological interventions are of limited efficacy. This study explored if metacognitive therapy (MCT); a brief transdiagnostic psychological intervention was potentially efficacious and could be delivered effectively to adult cancer survivors with psychological morbidity.

**Methods:** An open trial with 3- and 6-month follow-up evaluated the treatment effects of MCT in 27 consecutively referred individuals to a clinical psychology health service specializing in psycho-oncology. Each participant received a maximum of six 1-hour sessions of MCT. Levels of anxiety, depression, fear of cancer recurrence, post-traumatic stress symptoms, health related quality of life, and metacognitive beliefs and processes were assessed using self-report questionnaires.

**Results:** MCT was associated with statistically significant reductions across all outcome measures which were maintained through to 6-month follow-up. In the ITT sample on the primary treatment outcome measure, the Hospital Anxiety and Depression Scale-Total, 59% of participants met recovery criteria at post-treatment and 52% at 6-month follow-up, respectively. No participants significantly deteriorated. In the completer sample (*N* = 20), 80% recovered at post-treatment and 70% at 6-month follow-up. MCT was acceptable to patients with approximately 75% of patients completing all treatment sessions.

**Conclusion:** MCT, a brief transdiagnostic psychological intervention can be delivered effectively to a heterogenous group of cancer survivors with promising treatment effects. Examining the efficacy of brief MCT against the current gold standard psychological intervention would be a valuable advance toward improving the quality of life of cancer survivors.

## Introduction

The incidence of cancer in the United Kingdom is projected to increase by 2% over the next 15 years with survival rates also increasing. It is estimated that survival rates have doubled over the past 40 years with a ten-year survival rate of approximately 50% (Cancer Research UK, 2017) in 2016, there were an estimated 15.5 million cancer survivors which is expected to increase to 20.3 million by 2026 (National Cancer Institute, 2018). Psychological morbidity is common in cancer survivors. Approximately 25% of cancer survivors have clinically significant levels of anxiety and depression that could benefit from treatment (Hoffman et al., 2009). Posttraumatic stress disorder symptoms are common in cancer survivors with estimates ranging from 6 to 45% (Swartzman et al., 2017). Fear of cancer recurrence (FCR) is highly prevalent, a systematic review concluded that almost 60% of cancer survivors experience debilitating FCR (Simard and Savard, 2015). Psychological morbidity adversely impacts ongoing cancer care by reducing attendance at follow up screening appointments (DiMatteo et al., 2000; Thewes et al., 2014), health related quality of life (LeMasters et al., 2013) and increases healthcare costs (Carlson and Bultz, 2004; Jansen et al., 2016) and use of healthcare services (Elliott et al., 2011).

The substantial prevalence and associated problems with psychological morbidity in cancer survivors requires effective interventions. Unfortunately, highly efficacious psychological interventions are unavailable (Rehse and Pukrop, 2003; Osborn et al., 2006; Faller et al., 2013). The most widely evaluated and recommended psychological intervention is cognitive behavioral therapy (CBT) but it may be that core components of CBT; labeling cognitive distortions and reality testing negative automatic thoughts (NATs) are clinically limited where NATs will frequently reflect accurate thoughts about cancer recurrence and morbidity (Greer et al., 2010; Cook et al., 2015b). An intervention which does not need to focus on the content of cognition i.e., NATs, but instead focuses on core psychological processes underpinning psychological morbidity may be more efficacious for cancer survivors.

Metacognitive therapy (MCT; Wells, 2009) offers an alternative psychological approach to the treatment of psychological morbidity in cancer survivors. MCT is derived from a trans-diagnostic theory of psychopathology, the Self-Regulatory Executive Function (S-REF) model (Wells and Matthews, 1994, 1996). The model states that psychological morbidity becomes persistent when people use the cognitive-attentional syndrome (CAS) in response to unwanted thoughts. The CAS has three broad main components; (i) perseveration (worry, rumination, over-analyzing, repeatedly questioning one’s thoughts); (ii) attentional strategies (a heightened focus on possible signs of threat which can be internal e.g., signs of anxiety or external e.g., reminders of cancer); and (iii) unhelpful coping strategies (e.g., searching the internet for positive outcomes by cancer survivors, avoidance of reminders of cancer).

The S-REF model states that perseveration is guided by positive metacognitive beliefs about the helpfulness of worry and rumination: e.g., “worry will help me be better prepared,” “worry will ensure that I complete my daily tasks.” Unfortunately, worry and rumination achieve the opposite, because the person experiences more negative thoughts and views more situations as potentially dangerous. The individual repeatedly acts as if unwanted negative thoughts are meaningful which leads to the development of an inflexible way of responding to thoughts. A more flexible response style can help to alleviate perseveration. Similarly, the S-REF model specifies that threat monitoring (e.g., scanning for symptoms or for negative thoughts) is determined by positive metacognitive beliefs. More specifically, a person comes to believe that scanning the environment or one’s mind and/or body for symptoms will reduce distress whereas it leads to the persistence of threat and distress. Furthermore, negative metacognitive beliefs about the uncontrollability and danger of worry sustain and increase worry. Modifying negative metacognitive beliefs is fundamentally important in the S-REF model because, if patients believe that worry is uncontrollable, they will not attempt to control it. Therefore, it is possible that through targeting metacognitive beliefs and processes rather than cognitive content, MCT offers a particularly close “fit” with the needs of cancer survivors indicating potential for greater efficacy (McNicol et al., 2013).

The development of MCT for psychological morbidity in cancer is evolving with encouraging evidence for the explanatory and therapeutic utility of MCT. There is increasing evidence for the role of metacognitive beliefs and processes in emotional distress in cancer survivors from cross-sectional and prospective studies (Thewes et al., 2013; Cook et al., 2014, 2015a,b; Butow et al., 2015; Fisher et al., 2018) and in adult cancer patients undergoing chemotherapy (Quattropani et al., 2016, 2017). There have been two tests of the potential efficacy of MCT in cancer survivor. First, an open trial of MCT for emotional distress in adolescent and young adult cancer survivors found clinically significant reductions in anxiety, depression and posttraumatic stress symptoms (Fisher et al., 2015). Second, a multiple baseline study of MCT in four adult cancer survivors (Fisher et al., 2017) reported substantial reduction in anxiety, depression and FCR over six one-hour sessions These studies illustrate that MCT can rapidly alleviate psychological morbidity in cancer patients but before progressing to a randomized controlled trial, further evidence of the potential efficacy and feasibility of delivering MCT is required. The present study therefore examined if MCT delivered over six one-hour individual treatment sessions would result in clinically significant improvements in anxiety, depression, posttraumatic stress symptoms, fear and cancer recurrence and overall quality of life immediately following treatment and over a 6-month follow-up period. The study also examined if MCT would be associated with reductions in the metacognitive beliefs and processes.

## Materials and Methods

### Design

An open trial with follow-up at 3 and 6 months evaluated the potential efficacy of brief MCT for adult survivors of cancer experiencing emotional distress. Data was also gathered on recruitment and retention rates. All participants gave written informed consent in accordance with the Declaration of Helsinki. Ethical approval was provided by the National Health Service North West Research Ethics Committee (reference 15/NW/0820).

### Participants and Procedure

Potentially suitable participants were identified from consecutive referrals to an adult clinical heath psychology service which specializes in psychological interventions for cancer patients. Those patients with elevated scores on the Hospital Anxiety and Depression Scale (HADS; Zigmond and Snaith, 1983) and indicated a willingness to be approached for possible participation in an intervention were provided with an information sheet about the study. Those patients were contacted and invited to attend an assessment appointment to determine their suitability for inclusion. Following the informed consent procedure, clinical and demographic data was obtained by interview and participants completed a range of questionnaires assessing the severity of psychological morbidity (see section on measures). Participants also completed all questionnaires at post-treatment, and again at 3- and 6-month follow-up. All questionnaires were returned to an independent assessor who scored and entered the data.

Twenty seven cancer survivors participated in the study and met the following inclusion criteria: (i) a score of > 15 on the Hospital Anxiety and Depression Scale-Total (HADS-T); (ii) had been diagnosed with cancer ≥ 6 months previously; (iii) were aged 18 years or over; (iv) had completed acute medical treatment for cancer (i.e., chemotherapy, radiotherapy, surgery); (v) were not receiving concurrent psychological treatment; (vi) were not actively suicidal; (vii) reported no current substance use; (vii) were not experiencing a psychotic or organic illness; (viii) were free from psychotropic medication or has been on a stable dose for at least 8 weeks; and (ix) were able to speak and understand English.

### Intervention

Metacognitive therapy was delivered over a maximum of 6 individual face-to face sessions that were 45–60 min in duration. The intervention followed a manualized protocol (Wells, 2009). As the intervention was transdiagnostic, MCT followed the same protocol for each patient in the study regardless of symptom presentation. In session 1, the formulation template used when treating depression served as the basis for the development of an idiosyncratic case formulation for each participant, thus following the approach adopted in previous evaluations of MCT for cancer survivors (McNicol et al., 2013; Fisher et al., 2015, 2017). The next step in treatment is socialization which proceeds by sharing the case formulation and by Socratic Questioning to help the patient understand that each aspect of the CAS and several types of metacognitive beliefs are maintaining emotional distress. MCT then focuses on modifying negative beliefs about uncontrollability of rumination/worry through training in detached mindfulness (DM) and in rumination/worry postponement (Wells, 2009). Patients are helped to understand how naturally occurring thoughts (e.g., “I’m useless,” “What if my cancer comes back?,” “My family will not be able to cope”) do not necessarily lead to perseveration.). Rumination/worry postponement is a behavioral experiment to challenge the negative metacognitive belief that perseveration is an uncontrollable process. Positive metacognitive beliefs about the helpful nature of worry/rumination and the other unhelpful coping responses are also highlighted to the patients and addressed. Final sessions address relapse prevention and involve modifying remaining use of the “cognitive attentional syndrome,” reviewing any remaining conviction in positive and negative metacognitive beliefs and consolidating and alternative ways of responding to negative thoughts. Three therapists delivered MCT (PF, AB, and LF). Supervision was provided by PF on a weekly basis.

### Measures

#### Hospital Anxiety Depression Scale (HADS; Zigmond and Snaith, 1983)

The HADS is a 14-item self-report questionnaire measuring anxiety and depression (seven items each) over the past week. Each item is rated on a 4-point scale (0–3). Scores for each subscale range from 0 to 21 with higher scores reflecting more sever anxiety or depression. Scores of 11 or more on each of the subscales indicate caseness. Combining the two subscales provides a measure of emotional distress. The HADS-Total is the “gold standard” outcome measure for evaluating the efficacy of interventions on emotional distress in cancer populations, and has excellent psychometric properties (Luckett et al., 2010).

#### Impact of Events Scale-Revised (IES-R; Weiss, 2007)

The IES-R is a 22-item self-report questionnaire measuring trauma-related symptoms The total scale score ranges from 0 to -88 with higher scores indicative of more severe trauma symptoms. A total score of ≥ 33 indicates a probable diagnosis of PTSD (Weiss, 2007). The IES-R is validated for use in cancer populations with good psychometric properties (Salsman et al., 2015).

#### Fear of Cancer Recurrence Inventory (FCRI; Simard and Savard, 2009)

The FCRI is 42-item self-report questionnaire assessing 7 aspects of FCR. Each item is rated on a 5-point scale (0–4). A total score for the FCRI is obtained by summing scores on the 7 subscales, with higher scores indicating greater severity (range 0–168). The FCRI is the most validated measure of FCR across a wide range of cancer types (Simard and Savard, 2009).

#### Functional Assessment of Cancer Therapy-General (FACT-G; Cella et al., 1993)

The FACT-G is a 27 item self-report questionnaire that measures four domains of health-related quality of life (HRQOL). Each item is rated on a 5-point scale from 0 (not at all) to 4 (very much). The FACT-G total score ranges from 0 to 108 with higher scores indicating a better HRQOL. The FACT-G has been used extensively in mixed cancer populations and has excellent psychometric properties (Brucker et al., 2005).

#### Metacognitions Questionnaire-30 (MCQ-30; Wells and Cartwright-Hatton, 2004)

The MCQ-30 measures 5 domains of metacognition by 30 items. Participants rate the extent to which they “generally agree” with statements presented on a 4-point scale from 1 (do not agree) to 4 (agree very much), providing total scores for each subscale ranging from 6 to24. Higher scores indicate greater conviction in metacognitive beliefs. The MCQ-30 assesses: (1) positive beliefs about worry, (2) negative beliefs uncontrollability and danger of worry, (3) cognitive confidence, (4) beliefs about the need to control thoughts, and (5) cognitive self-consciousness. The MCQ-30 has been validated for use in cancer patients (Cook et al., 2014).

#### Cognitive Attentional Scale-1 (CAS-1; Wells, 2009)

The CAS-1 is a 10 item self-report questionnaire that assesses metacognitive processes and beliefs. Items 1 to 6 assess the fundamental components of the CAS (perseverative thinking, threat monitoring and unhelpful coping strategies) Each item is rated on a 10-point scale from 0 (none of the time) to 100 (all the time). Items 7 to 10 assess metacognitive beliefs and are not reported in the present study. To provide an overall measure of the CAS, the 6 items were summed and divided by the number of items. The same method has been used previously (Fisher et al., 2016; Heffer-Rahn and Fisher, 2018).

### Statistical Analyses

Intention to treat (ITT) analyses were used to determine the potential efficacy of brief MCT for emotional distress in cancer survivors. Missing data for the non-completers in the study were replaced by using the last observation carried forward (LOCF) method. The LOCF has been considered a conservative approach when evaluating treatment outcomes in open trials. Treatment effects across time (pre-treatment, post-treatment, and 3- and 6-month follow-up) were assessed with repeated-measures analysis of variance (ANOVA); the Greenhouse–Geisser correction was applied when the assumption of sphericity was violated. Main effects were followed by Bonferroni-adjusted pairwise comparisons for each outcome measure. Within group effect sizes were calculated using Cohen’s *d* to assess the magnitude of treatment effects from pretreatment to post-treatment and from pre-treatment to both 3- and 6-month follow-ups. To determine the clinical significance of treatment effects the methodology developed by Jacobson et al. (1984) and Jacobson and Truax (1991) was applied to the HADS-Total. Each patient can be allocated to one of four treatment outcomes: reliable deterioration, no change, reliable improvement, or recovered. The first three outcomes are calculated using from the Reliable Change Index (RCI), which determines whether the magnitude of change is statistically significant. Data to calculate the RCI was drawn from a large non-clinical sample (Crawford et al., 2001). The cut-off score for the HADS-Total was ≤ 13 determined using “criterion a” To be classified as recovered, patients must demonstrate reliable change and their post-treatment or follow-up scores must be below the cut off score. The data were analyzed using SPSS version 24.

## Results

### Participant Characteristics

Forty-three consecutive referrals were identified as potentially eligible. There were 16 patients who did not enter the study; 10 did not wish to participate, 3 did not attend the assessment interview 1 patient did not have a have a cancer diagnosis, 1 patient did not meet the threshold for severity of distress with a HADS-T score of less than 16 and 1 patient had a recurrence of cancer.

Twenty-seven patients began the trial of whom 20 completed treatment; a completion rate of 74%. Of the seven patients who did not complete the six sessions of MCT; three patients attended only one session, two patients 2 sessions, one patient 3 sessions and the final patient attended 4 sessions but sporadically and decided that it was not feasible to continue therapy. Reasons for non-completion were; one patient was hospitalized for cancer recurrence, one participant stopped therapy to be able to provide full time care for a relative, 2 participants did not wish to undertake psychological therapy and 3 patients dropped out without providing a reason. The demographic and clinical characteristics of the sample shown in Table 1. It is notable that 96% of the sample met caseness for anxiety with 93% also scoring above the clinical cut-off for PTSD. Additionally, 8 of the 27 patients had experienced a cancer recurrence, none of these patients discontinued MCT.

**Table 1:** Participant characteristics.

	Mean (*SD*)	Range
Age	51.15 (11.67)	29–67
Age at time of cancer diagnosis	46.71 (10.99)	28–64
Months since completion of acute medical treatment	25.81 (27.93)	3–142
	
	*N*	
	
*Gender*		
Female	23	
Male	4	
*Ethnicity*		
White Caucasian	26	
Asian	1	
*Cancer Diagnosis*		
Breast	13	
Hematological	6	
Ovarian	3	
Sarcoma	2	
Colorectal	1	
Ocular	1	
Lung	1	
*Cancer Treatment*		
Chemotherapy, radiotherapy, and surgery	8	
Chemotherapy plus surgery	5	
Chemotherapy alone	4	
Surgery alone	3	
Chemotherapy, plus radiotherapy	2	
Radiotherapy plus surgery	1	
Radiotherapy alone	1	
Other/not reported	3	
*Employment Status*		
Employed	13	
Unemployed	14	
*Education Level*		
School level or higher No qualifications	26 1	
*Relationship Status*		
Married/cohabiting	11	
Live alone	16	
*Psychotropic Medication*		
Current taking	11	
Previously taken	5	
Never taken	11	
*Previous Psychological Treatment*		
Yes	17	
No	10	
*Distress Outcomes*		
Anxiety (HADS-A > 11)	26 (96%)	
Depression (HADS-D > 11)	12 (44%)	
PTSD symptoms (IES-R > 33)	25 (93%)	


### Treatment Effects

There were significant main effects of time on all outcome measures (Table 2). Follow-up Bonferroni pairwise comparisons demonstrated significant differences from pre-treatment to post-treatment, and from pre-treatment to 3-and 6-month follow up on all outcome measures indicating that treatment effects were maintained. Overall, there was significant improvement across all symptom and quality of life measures and significant reductions in metacognitive beliefs (MCQ-30) and processes (CAS-1).

**Table 2:** Means, standard deviations (in parentheses) and repeated measures analysis of variance for outcome measures: Intention-to-treat sample (*n* = 27).

Measure	Pre-treatment	Post-treatment	3-month follow-up	6-month follow-up	*F* (*df*)	
HADS -Total	25.04 (5.65)	12.70 (9.61)	13.00 (9.99)	12.67 (10.12)	39.76 (2.15, 56.05)	*p* < 0.0001
HADS - Anxiety	14.44 (3.51)	7.85 (5.14)	7.96 (5.49)	7.52 (5.44)	32.85 (2.21, 57.30)	*p* < 0.0001
HADS - Depression	10.74 (3.77)	4.81 (4.79)	5.04 (4.89)	5.15 (5.23)	31.60 (2.03, 52.71)	*p* < 0.0001
IES-R -Total	53.15 (16.43)	26.04 (26.93)	27.92 (26.64)	27.81 (25.35)	26.56 (2.32, 60.28)	*p* < 0.0001
FCRI- Total	108.29 (22.18)	59.59 (38.84)	63.37 (36.63)	63.81 (36.73)	34.42 (1.49, 38.48)	*p* < 0.0001
FACT-G-Total	54.33 (14.94)	76.87 (20.16)	74.55 (21.63)	74.94 (22.73)	31.09 (2.21, 57.43)	*p* < 0.0001
MCQ-30 Positive beliefs	11.74 (4.66)	8.22 (3.73)	8.29 (3.61)	8.33 (3.89)	9.47 (1.48, 38.53)	*p* < 0.001
MCQ-30 Negative beliefs	18.59 (3.27)	11.85 (5.23)	12.03 (5.21)	11.70 (5.04)	28.87 (1.78, 46.28)	*p* < 0.0001
MCQ-30 Cognitive confidence	15.74 (5.28)	11.41 (4.98)	12.48 (5.61)	11.77 (5.58)	13.35 (2.18, 55.06)	*p* < 0.0001
MCQ-30 Need for control	14.41 (4.38)	10.07 (4.73)	9.33 (4.72)	9.26 (4.77)	23.30 (1.40, 36.40)	*p* < 0.0001
MCQ-30 Cognitive self-consciousness	17.93 (3.98)	12.66 (6.09)	12.52 (4.87)	12.59 (5.15)	23.85 (2.27, 59.05)	*p* < 0.0001
CAS-1	55.25 (19.19)	20.06 (25.85)	20.86 (26.19)	24.32 (28.61	44.67 (2.18, 56.69)	*p* < 0.0001


*df, degrees of freedom; HADS, Hospital Anxiety and Depression Scale; IES-R, Impact of Event Scale-Revised; FCRI, Fear of Cancer Recurrence Inventory; FACT-G, Functional Assessment of Cancer Therapy-General; MCQ-30, Metacognitions Questionnaire-30; CAS-1, Cognitive Attentional Scale.*

### Effect Size Estimates

Within group effect sizes for the ITT sample are shown in Table 3. There are large pre to post-treatment effect sizes across all outcome measures (0.83–1.66). There are comparable effect sizes across all measures at both follow-up timepoints illustrating that the magnitude of treatment effects is maintained from post-treatment to 6-month follow-up.

**Table 3:** Within group effect sizes (Cohen’s *d*) for outcome measures at post-treatment and 3- and 6-month follow-up.

	Post-treatment	3-month follow-up	6-month follow-up
HADS-Total	1.56	1.48	1.51
HADS-Anxiety	1.49	1.41	1.51
HADS-Depression	1.37	1.31	1.23
IES-R Total	1.21	1.14	1.18
FCRI-Total	1.66	1.48	1.46
FACT-G-Total	–1.27	–1.09	–1.07
MCQ-30 Positive beliefs	0.83	0.83	0.79
MCQ-30 Negative beliefs	1.51	1.50	1.62
MCQ-30 Cognitive confidence	0.84	0.59	0.75
MCQ-30-Need for control	0.95	1.12	1.12
MCQ-30-Congnitive self-consciousness	1.02	1.22	1.16
CAS-1	1.55	1.49	1.27


*HADS, Hospital Anxiety and Depression Scale; IES-R, Impact of Event Scale-Revised; FCRI, Fear of Cancer Recurrence Inventory; FACT-G, Functional Assessment of Cancer Therapy-General; MCQ-30, Metacognitions Questionnaire-30; CAS-1, Cognitive Attentional Scale.*

### Clinically Significance of Treatment

In the ITT sample, most participants were recovered on the HADS-Total at post-treatment and across the follow-up period. In terms of the proportion of patients that responded to treatment, 81% were improved at post-treatment and 74% at 6-month follow-up. Examination of the recovery rates for those patients that completed treatment shows recovery rates of 80% at post-treatment and 70% at 6-month follow-up. A summary of the clinical significance of treatment outcomes is shown in Table 4.

**Table 4:** Clinical significance outcomes on HADS-total.

	Post-treatment			3-month follow-up				6-month follow-up		
	No change	Improved	Recovered	Deteriorated	No change	Improved	Recovered	No change	Improved	Recovered
ITT	5	5	17	1	4	8	14	7	5	15
(*n* = 27)	19%	19%	62%	5%	25%	17%	58%	26%	19%	56%
Completers	1	3	16	1	0	6	13	3	3	14
(*n* = 20)	20%	0%	80%	5%	0%	30%	65%	15%	15%	70%


*ITT: intention to treat sample; Completers: treatment completers sample.*

## Discussion

This study provides further support for the potential of brief MCT to alleviate psychological morbidity in cancer survivors. Following six 1-hour sessions of MCT, there were significant reductions in anxiety depression, post-traumatic stress symptoms, FCR and improvements in quality of life. There were also significant reductions in metacognitive beliefs and the CAS as predicted by the metacognitive model (Wells and Matthews, 1994, 1996). Treatment gains were sustained across all measures of psychological morbidity and metacognitive beliefs and processes through to 6-month follow-up. The practical significance as opposed to the statistical significance of the results was assessed using the Jacobson approach to clinical significance. In those patients who completed brief MCT, there were very high recovery rates on the primary outcome variable assessing the severity of general distress; 80% of patients were recovered following six one-hour sessions of individually delivered MCT. The recovery rate of 70% at 6-month follow-up suggests that the effects of the intervention persist beyond treatment completion. Brief MCT appeared acceptable to cancer survivors with approximately 75% of participants starting treatment completed treatment. It is possible that the treatment completion rate can be improved and early drop-outs from treatment prevented by ensuring patients are more effectively socialized to the aims of MCT.

The within group effect sizes on FCR provide the opportunity to benchmark the effects of brief MCT with those reported in recent randomized controlled trial evaluating an integrative approach for FCR. The psychological treatment in the trials conducted by Butow et al. (2017) evaluated an intervention (ConquerFear) based on the treatment components drawn from three theoretical frameworks; common sense model (Leventhal et al., 1992) the self-regulatory model (Wells and Matthews, 1994) and relational frame theory (Hayes et al., 2006). Although the ConquerFear intervention was more efficacious than an attention control condition, the within group effect size for FCR from pre to post-treatment was 0.77. This compares to a within group effect size of 1.66 in the present study. Although, the present study had a much smaller sample size thereby limiting the generalizability of this finding. However, unlike the ConquerFear study, our open trial included participants with depression and severe trauma symptoms indicative of PTSD. Developing specific interventions for each aspect of psychological morbidity for cancer survivors may be unnecessary and integrating treatment components from theoretically inconsistent models could “dilute” treatment efficacy and compromise therapist training (Wells and Fisher, 2015; Byrne et al., 2018).

The present open trial is a valuable step in the translation of MCT from adult mental health populations to cancer survivors and is following the recommended framework for translating psychological interventions to a new population (Craig et al., 2008). The limitations of open trials are well known but should not undermine their place in treatment development research (Craig et al., 2008). No data was collected on either treatment adherence or therapist competency beyond that achievable through weekly supervisory sessions. Subsequent studies should include independent assessment of both treatment adherence and therapist competency to increase confidence in the conclusions drawn and that any treatment effects were attributable to MCT.

A comparatively small sample was used, but the sample appeared representative of cancer survivors referred to the clinical health psychology service. Other limitations include the lack of ethnic diversity and that most of the sample were female, thereby compromising external validity. Treatment outcome was assessed exclusively by self-report questionnaires in the present study. Although exclusive reliance on self-report questionnaires could be considered a methodological weakness, the study was not focused on changes psychiatric diagnosis, rather the study was designed to measure general distress for which the “gold standard” outcome measure for evaluating the efficacy of interventions on emotional distress in cancer was used (Luckett et al., 2010).

Overcoming other limitations of open trials can be achieved through conducting randomized controlled evaluation. It would be valuable to assess the hypothesized mechanisms of change in the context of an RCT against the current recommended treatment approaches, it may be that the treated patients who recover change to most on metacognitive variables regardless of the treatment received. There were statistically significant reductions in all metacognitive beliefs and the CAS over treatment, which were maintained through to the 6-month follow up assessment. This study adds to the extant literature that MCT has the potential to be an efficacious psychological intervention for adult cancer survivors. Given the limited outcomes of currently available interventions, there is an obvious need to conduct a controlled evaluation of the potential of brief MCT to alleviate psychological morbidity in cancer survivors.

## Author Contributions

PF and PS designed the study. AB, LF, and PF were the therapists. PF drafted the manuscript. GA and HU recruited and assessed participants at intake and following treatment. All authors have contributed to drafting and revising the manuscript and approved its submission.

## Conflict of Interest Statement

The authors declare that the research was conducted in the absence of any commercial or financial relationships that could be construed as a potential conflict of interest.
